# Supplementary material for: Evolutionary Modes of wtf Meiotic Driver Genes in Schizosaccharomyces pombe
Source: Genome Biol Evol. 2024 Oct 11;16(10):evae221. doi: 10.1093/gbe/evae221 (PMC11497594; doi:10.1093/gbe/evae221)
Supplement: evae221_Supplementary_Data [file evae221_supplementary_data.zip › Sup_figures_2024Oct09.pdf]

Supplementary Information

Evolutionary modes of *wtf* meiotic driver genes in *Schizosaccharomyces pombe*

Yan-Hui Xu, Fang Suo, Xiao-Ran Zhang, Tong-Yang Du, Yu Hua, Guo-Song Jia, Jin-Xin Zheng, Li-Lin Du

TABLE OF CONTENTS

Supplementary Figure 1 2
Supplementary Figure 2 4
Supplementary Figure 3 6
Supplementary Figure 4 8
Supplementary Figure 5 10
Supplementary Figure 6 12
Supplementary Figure 7 13
Supplementary Figure 8 14
Supplementary Figure 9 15
Supplementary Figure 10 16

Supplementary Tables 1–8 are provided in a separate Excel file.

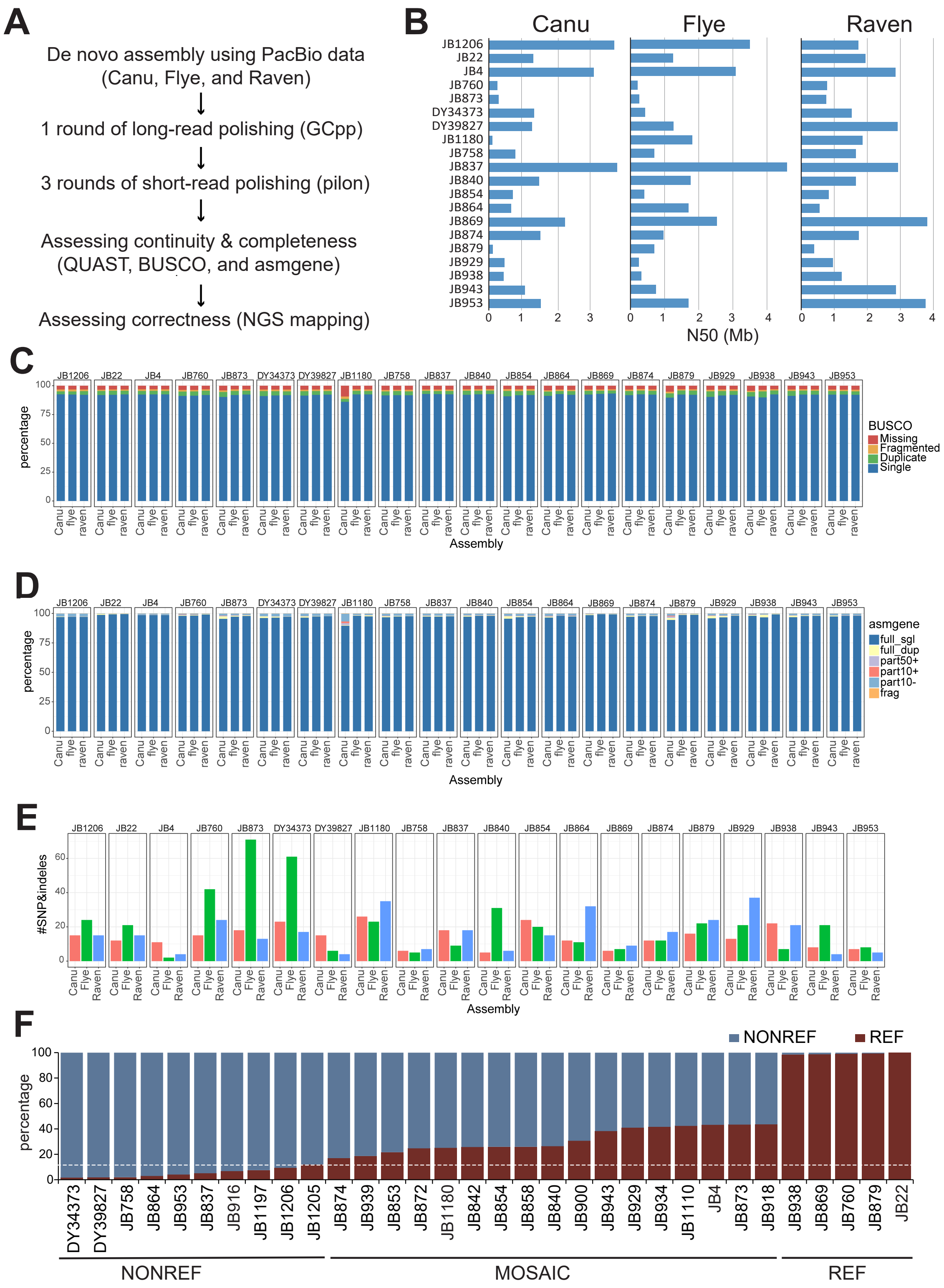

Supplementary Figure 1. Quality assessment of long-read-based genome assemblies and the lineage ancestry of the 32 isolates.

(A) Flowchart depicting the pipeline used in this study for de novo assembly of PacBio sequencing data of 20 *S. pombe* isolates and the subsequent assessment of assembly quality.

(B) N50 values (an indicator of assembly contiguity) of the 60 assemblies reported by QUAST.

(C) Assembly completeness assessment results reported by BUSCO.

(D) Assembly completeness assessment results reported by asmgene.

(E) Assembly correctness assessment by mapping Illumina sequencing reads to the assemblies and calling variants using GATK, Samtools, and DeepVariant. The barplot shows the number of SNPs and indels called by at least two variant calling tools.

(F) Lineage ancestry of the 32 isolates whose *wtf* genes have been comprehensively analyzed in this study. The sequence of a representative assembly for each isolate was divided into 20-kb windows. The number of SNPs relative to JB22 (a REF lineage isolate) was calculated for each window. The percentage of REF lineage sequence in each isolate was determined by dividing the number of windows containing no more than 20 SNPs by the total number of windows. The dashed line denotes 10% REF lineage sequence, a threshold for distinguishing pure-lineage isolates and mosaic isolates (Tusso *et al.* 2019).

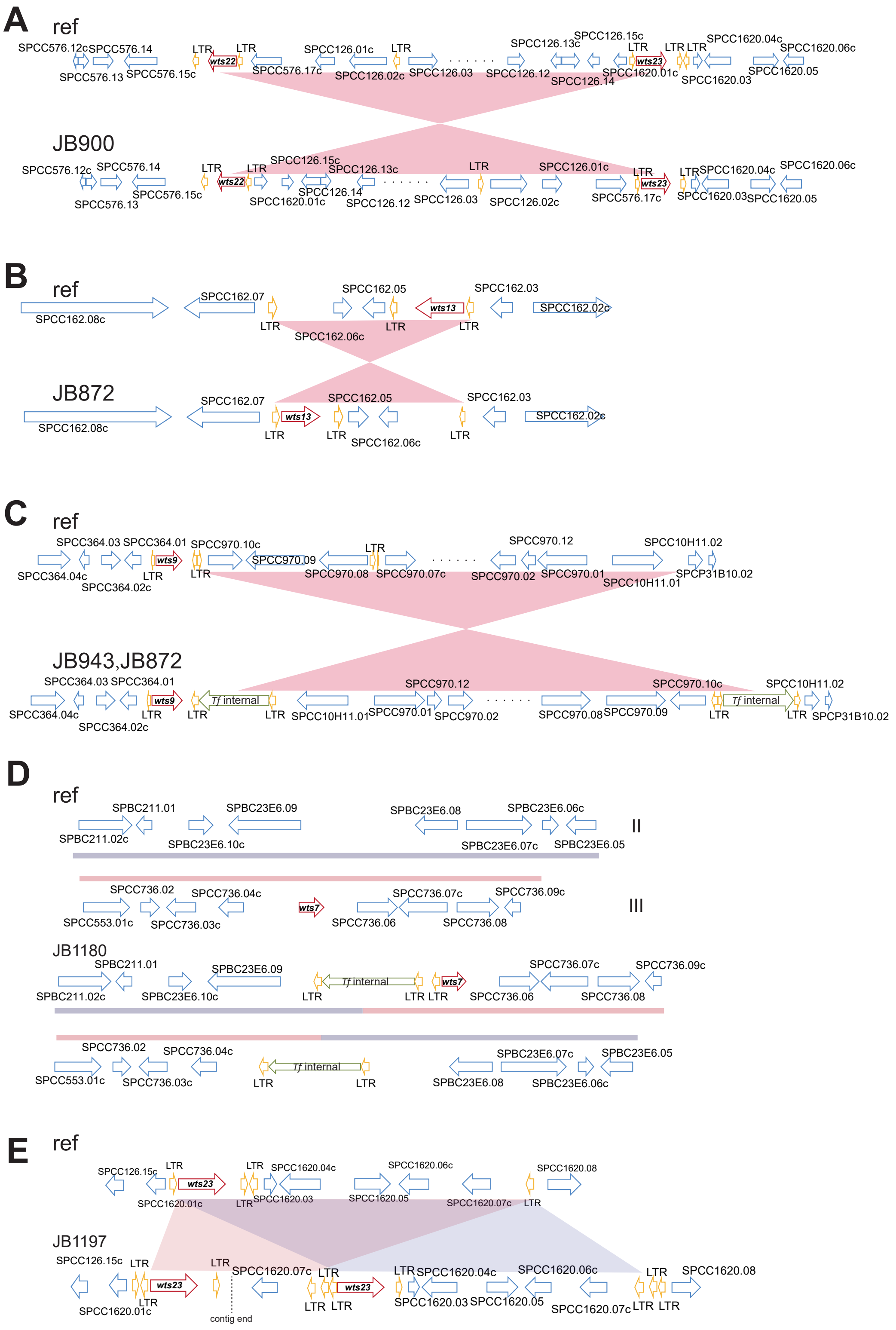

Supplementary Figure 2. Schematics of genome rearrangements altering the order of unique genes surrounding *wtf* genes.

(A) An inversion whose breakpoints fall into the gene bodies of *wts22* and *wts23* in JB900. This inversion was likely caused by recombination between these two *wtf* genes.
(B) An inversion altering the gene order surrounding *wts13* in JB872. This inversion was likely caused by recombination between solo LTRs.
(C) An inversion altering the gene order surrounding *wts9* in JB943 and JB872. This inversion was likely caused by recombination between full-length Tf transposons.
(D) A translocation altering the gene order surrounding *wts7* in JB1180. This translocation was likely caused by recombination between full-length Tf transposons.
(E) Duplication of *wts23* in JB1197. This duplication was likely caused by recombination between solo LTRs.

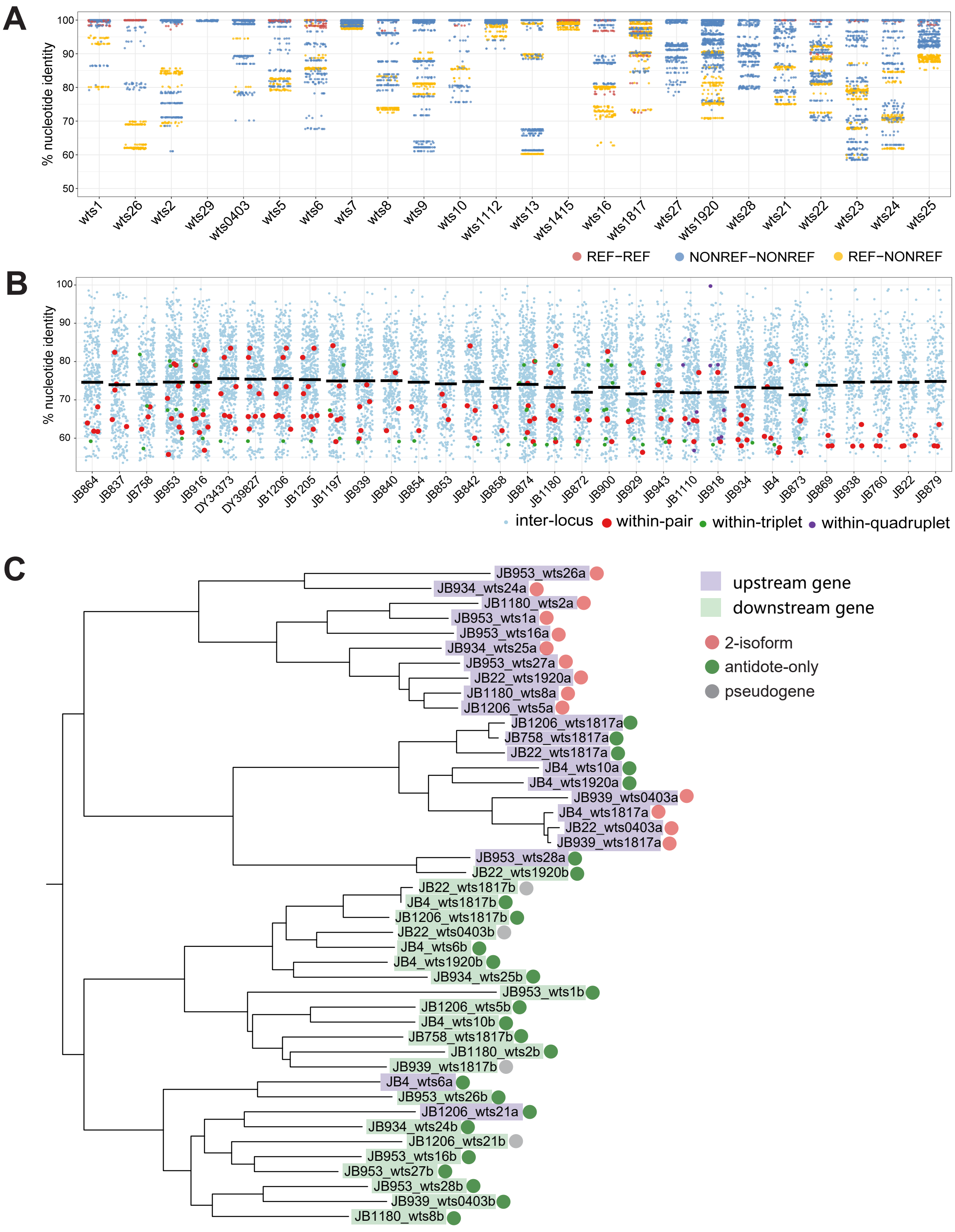

Supplementary Figure 3. Sequence diversity of *wtf* genes.

(A) Pairwise nucleotide identities of syntenic *wtf* genes in different isolates (inter-isolate comparisons). For genes located in multi-gene loci, comparisons were only made when

the syntenic loci contained the same number of *wtf* genes. Three different colors are used to distinguish comparisons between genes sharing the REF-lineage ancestry, between genes sharing the NONREF-lineage ancestry, and between genes having different lineage ancestry. Genes in loci with uncertain ancestry are not included in this analysis.

(B) Pairwise nucleotide identities of typical *wtf* genes in the same isolate. The median identities are represented by black horizontal lines. Different colors are used to distinguish comparisons between genes in different loci (inter-locus comparisons) and comparisons between genes in the same multi-gene locus (within-pair, within-triplet, and within-quadruplet comparisons).

(C) Phylogenetic relationship of 44 *wtf* genes from 22 representative gene pairs. These gene pairs were selected to maximize diversity. The gene names are shown with backgrounds of different colors to distinguish between upstream genes and downstream genes. Color-filled circles next to the gene names denote the functional type of genes (2-isoform, antidote-only, or pseudogene). The phylogenetic tree was manually rooted at the branch separating the two clusters to which most upstream genes and most downstream genes respectively belong.

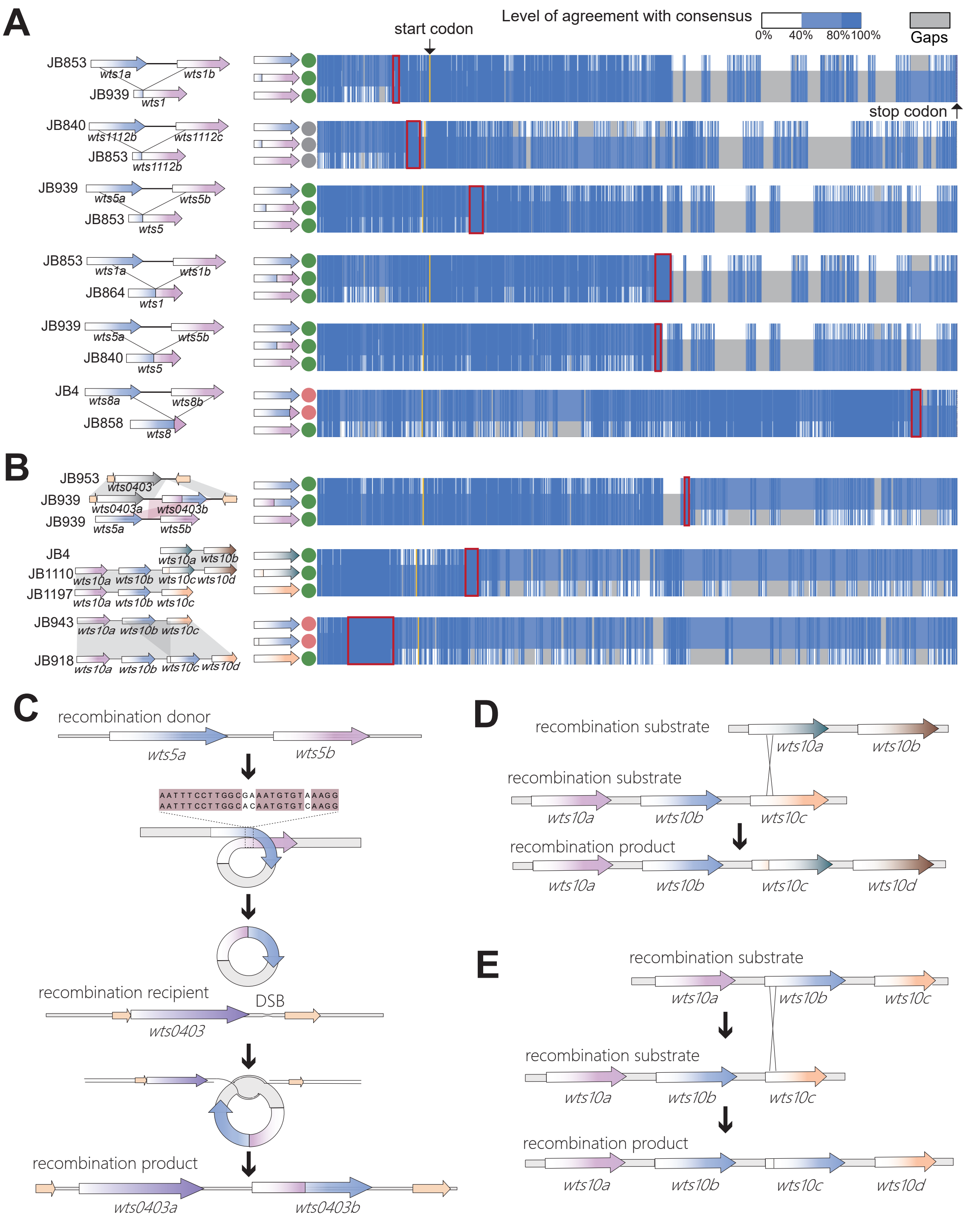

Supplementary Figure 4. Recent evolutionary events altering the number of *wtf* genes per locus.

(A) The 6 events decreasing the number of *wtf* genes per locus. Each event is illustrated

with a schematic on the left side, depicting two *wtf* genes in the ancestral allele in a representative isolate (top) and a *wtf* gene resulting from recombination of the two ancestral genes in the derived allele in a representative isolate (bottom). The two representative isolates in each case belong to the same phylogroup. The Jalview-generated alignment overview shown on the right side provides more details on how recombination occurs, with the recombination breakpoint highlighted by a red box. The alignment is of sequences starting from the conserved_up region and ending at the stop codon. The start codon of the antidote is highlighted in yellow in the alignment. The functional types of the genes are depicted using color-filled circles, as shown in Figure 2.
(B) The 3 events increasing the number of *wtf* genes per locus. The schematics on the left side are as follows: for the first event, the ancestral allele (top), the recombination product in the derived allele (middle), and the recombination donor (bottom) are shown; for the second event, two ancestral alleles are shown at the top and bottom, respectively, and the derived allele resulting from recombination of the two ancestral alleles is shown in the middle; for the third event, the ancestral allele is shown at the top, and the derived allele resulting from recombination of two copies of the ancestral alleles is shown at the bottom. The Jalview-generated alignment overview shown on the right side provides more details on how recombination occurs, with the recombination breakpoint highlighted by a red box. The alignment is of sequences starting from the conserved_up region and ending at the stop codon. The start codon of the antidote is highlighted in yellow in the alignment. The functional types of the genes are depicted using color-filled circles as in Figure 2.
(C-E) Schematics illustrating the detailed processes of the events shown in (B).

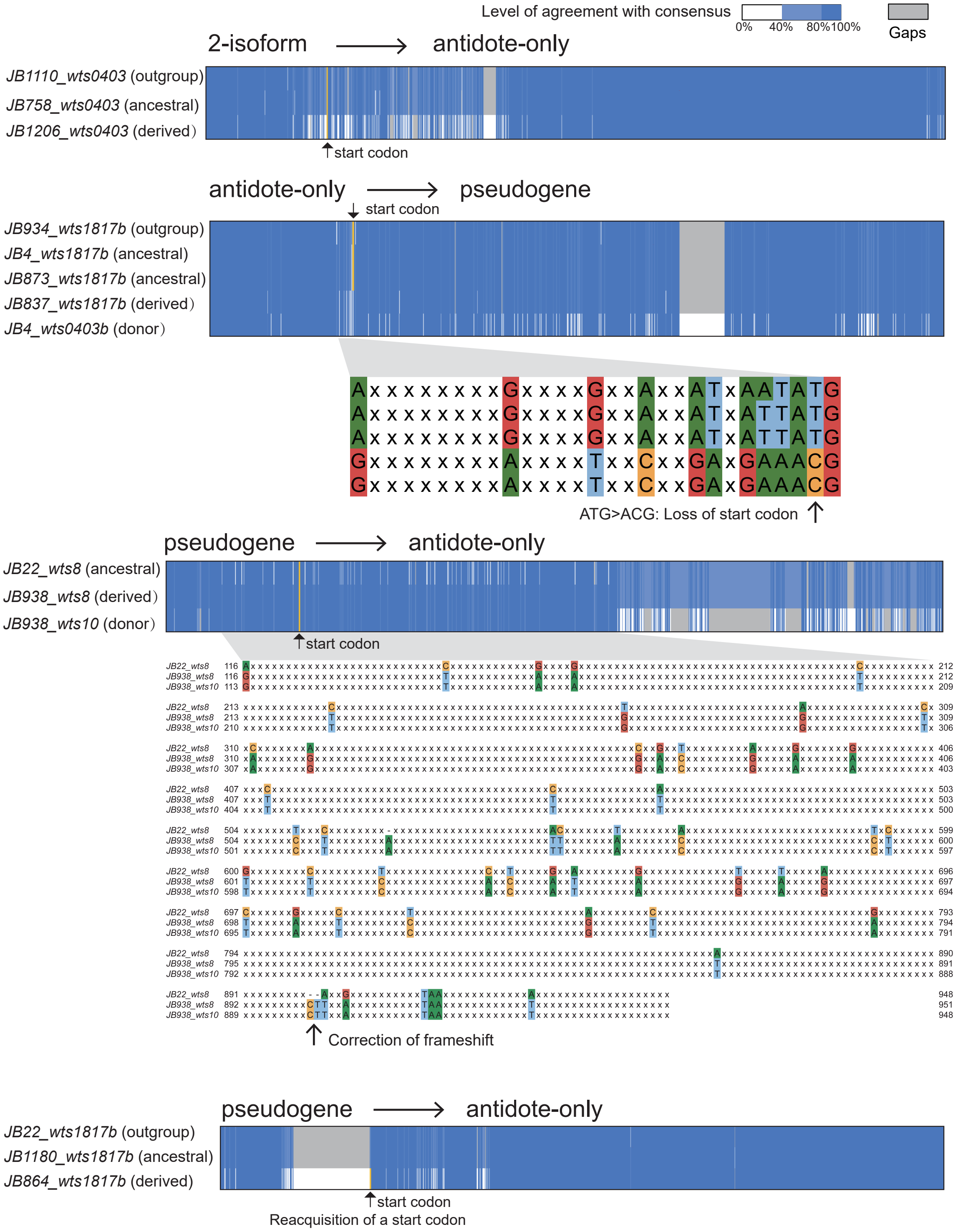

Supplementary Figure 5. Non-allelic gene conversion events changing the functional type of *wtf* genes. Jalview-generated alignment overviews illustrate where gene

conversion occurred. The alignment includes sequences starting from the conserved_up region and ending at the stop codon. The start codon of the antidote is highlighted in yellow in the alignment. In the case of two events, the nucleotide sequences in the regions that underwent gene conversion are shown in zoomed-out views of the alignments. In these zoomed-out views, identical nucleotides are represented as "x".

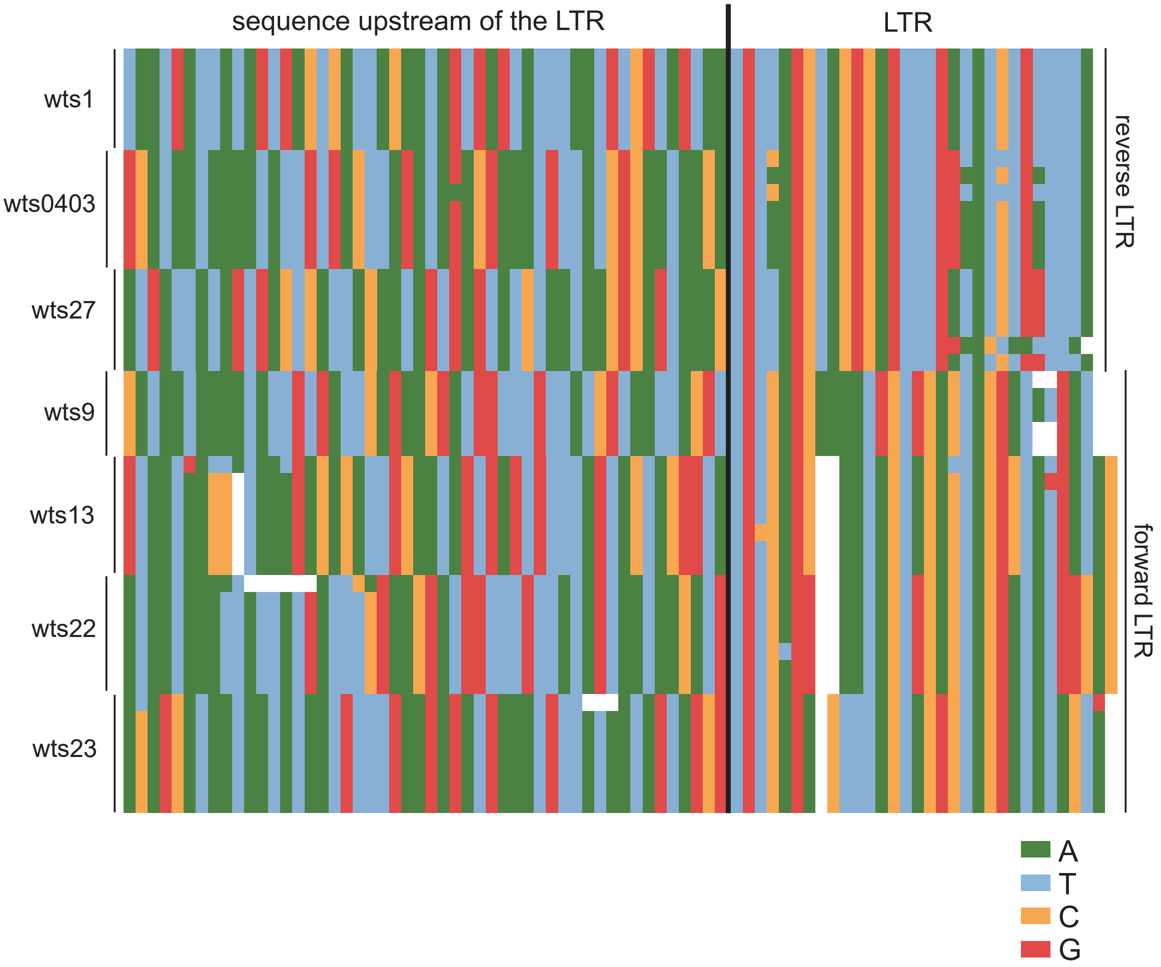

Supplementary Figure 6. Lack of inter-locus similarities among sequences on the *wtf*-gene-proximal side of the LTRs located downstream of a *wts* locus.

In the nucleotide sequence alignment, each sequence includes a 50-bp sequence upstream of the LTR and a 30-bp *wtf*-gene-proximal portion of the LTR. The sequences are from 7 representative isolates and are ordered from top to bottom as follows: JB22, JB1180, JB4, JB758, JB864, JB953, and JB872. For the *wts27* locus, there are only 6 sequences as JB22 does not harbor *wtf* genes at this locus. For the *wts1* locus, there are only 6 sequences due to the exclusion of the sequence JB758, in which an LTR is inserted within the 50-bp sequence. For the *wts9* locus, there are only 5 sequences due to the exclusion of the sequences in JB22 and JB872, because an LTR is inserted within the 50-bp sequence in JB22 and a full-length Tf transposon is inserted within the 50-bp sequence in JB872.

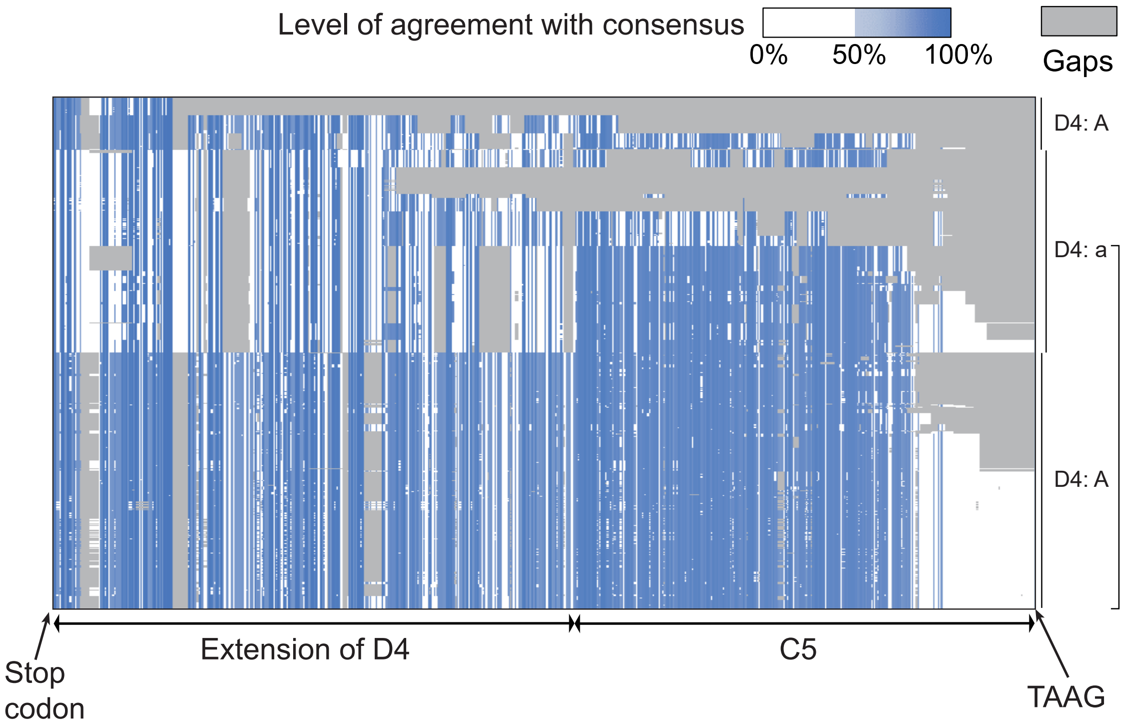

Supplementary Figure 7. Alignment of the conserved_down sequences of typical *wtf* genes.

The alignment is shown as a Jalview-generated overview. Gaps in the alignment are shown in dark grey. Nucleotides are colored according to the percentage agreement with the consensus. The sequence types of the D4 region are indicated by vertical lines on the right side of the alignment. The bracket on the right side denotes the sequences included in the alignment shown in Supplementary Figure 8.

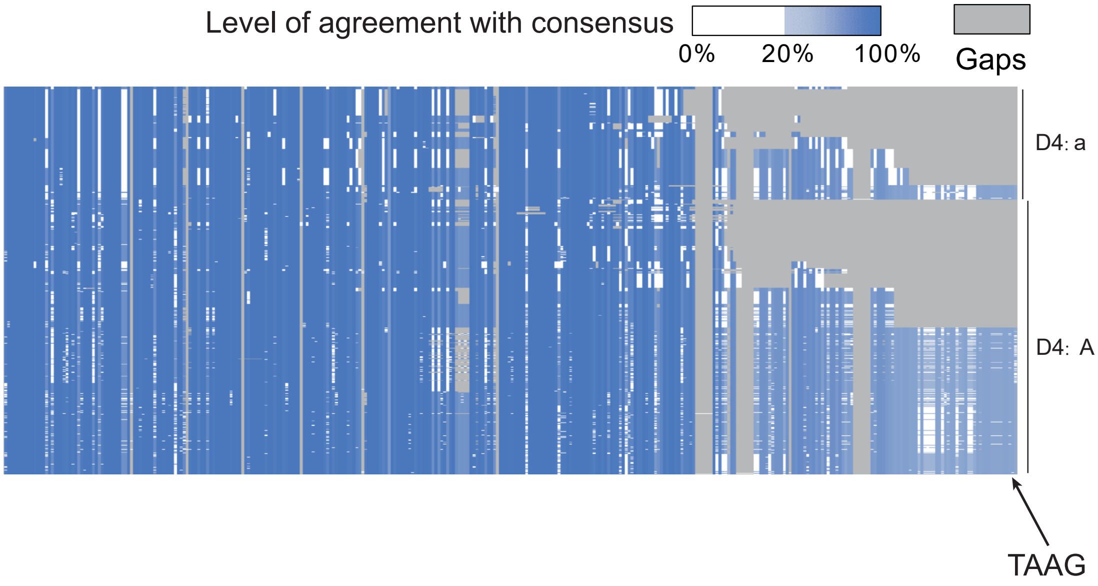

Supplementary Figure 8. Alignment of the C5 region of the conserved_down sequences of typical *wtf* genes.

The alignment is shown as a Jalview-generated overview. Gaps in the alignment are shown in dark grey. Nucleotides are colored according to the percentage agreement with the consensus. Sequences included in this alignment are those denoted by the bracket in Supplementary Figure 7. The sequence types of the D4 region are indicated by vertical lines on the right side of the alignment. Please note that the percentage agreement threshold for using blue color to indicate a match to the consensus is lower than that in Supplementary Figure 7.

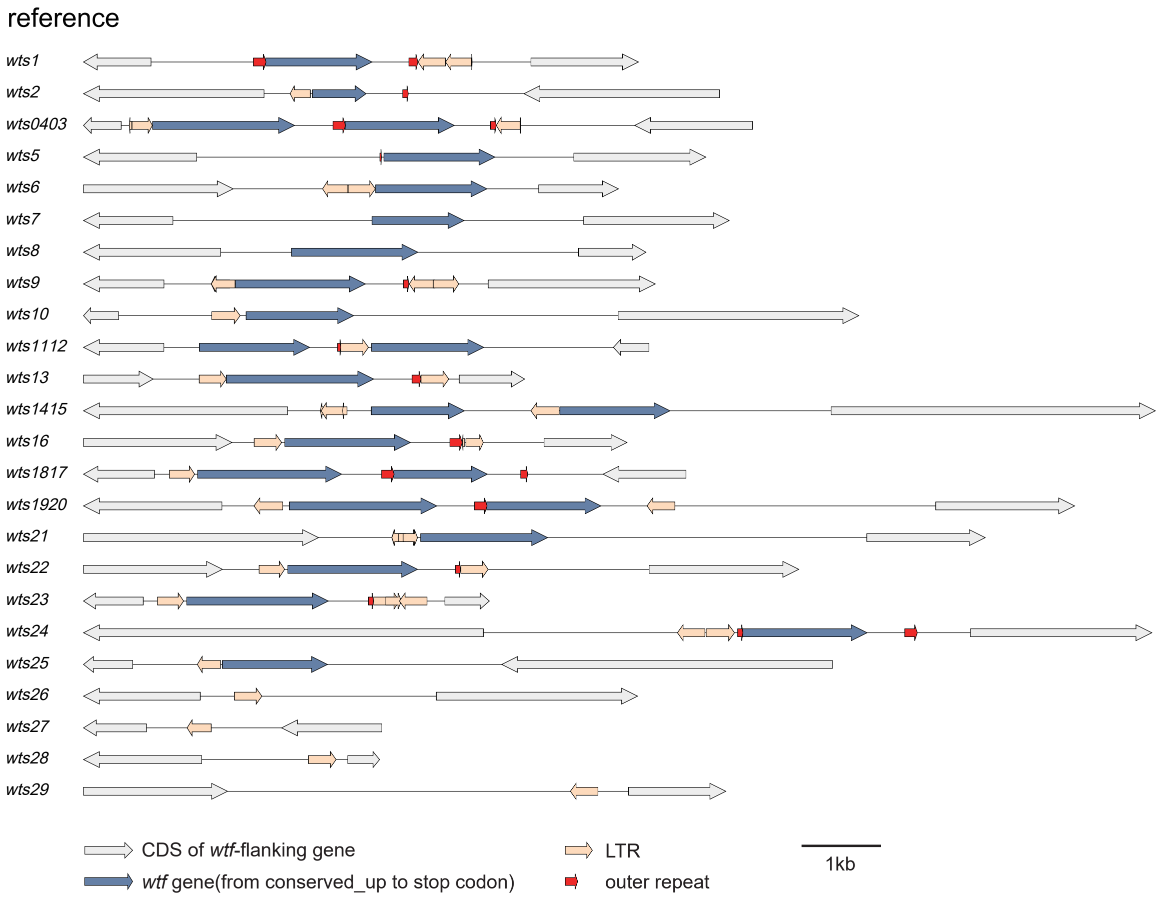

Supplementary Figure 9. Diagram depicting the outer repeats and *wtf*-flanking LTRs in the reference genome, which is representative of the genomes of REF lineage isolates.

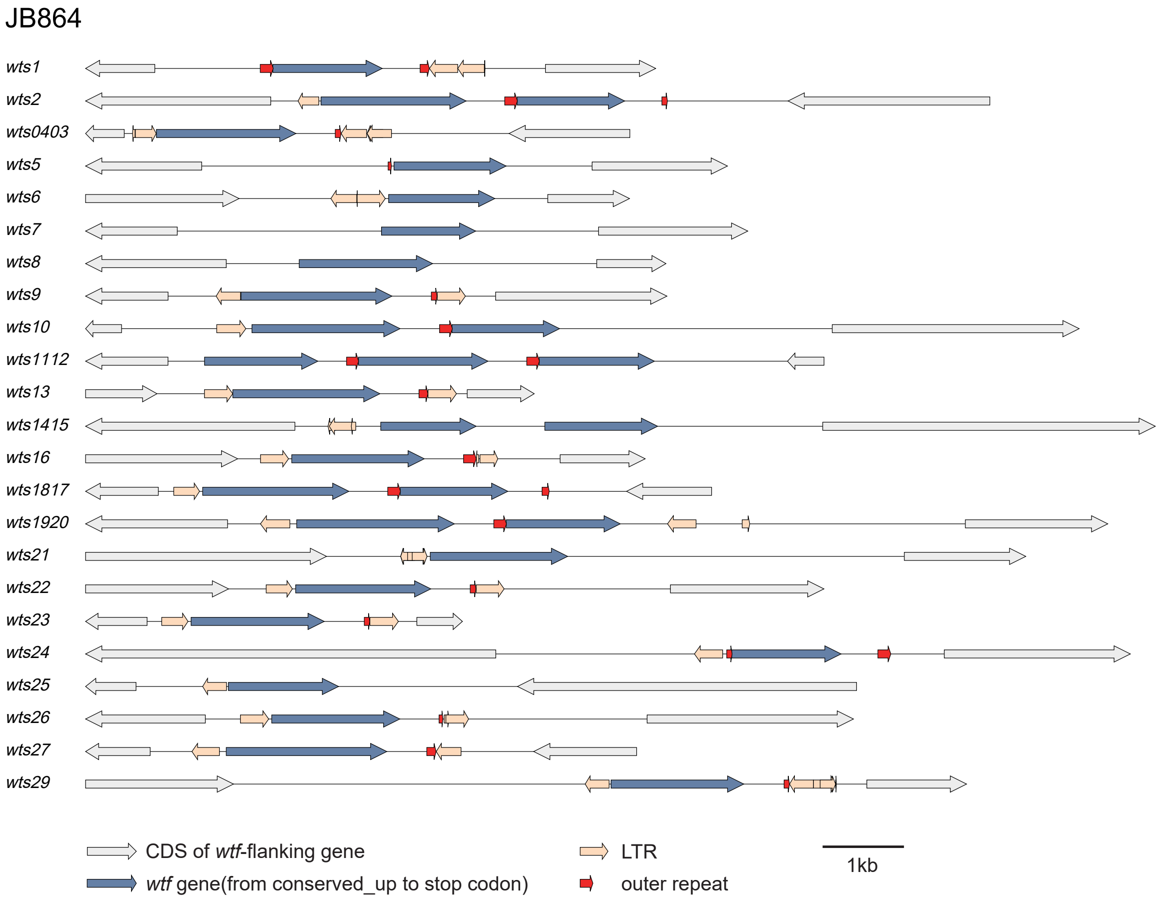

Supplementary Figure 10. Diagram depicting the outer repeats and *wtf*-flanking LTRs in the genome of JB864, which is representative of the genomes of NONREF lineage isolates. The *wts28* locus is not depicted because the exact sequence of the *wtf* gene at that locus is uncertain.
